# Preventive exercise intervention for trismus in head and neck cancer – a randomized study: Preventive exercise intervention for trismus

**DOI:** 10.1007/s00784-026-06838-3

**Published:** 2026-03-31

**Authors:** Nina Pauli, Lisa Tuomi, Ellen Lindell, Helen Larsson, Caterina Finizia

**Affiliations:** 1 Region Västra Götaland, Department of Otorhinolaryngology, Head and Neck Surgery, Sahlgrenska University Hospital, Gothenburg, Sweden; 2 Department of Otorhinolaryngology, Head and Neck Surgery, Institute of Clinical Sciences, Sahlgrenska Academy, University of Gothenburg, Gothenburg, S-41345 Sweden; 3 Institute of Neuroscience and Physiology, Speech and Language Pathology Unit, Sahlgrenska Academy, University of Gothenburg, Gothenburg, Sweden; 4 Region Västra Götaland, Department of Research, Education and Innovation, Södra Älvsborgs Hospital, Borås, Sweden; 5 Region Västra Götaland, Department of ENT & Maxillofacial Surgery, NAL Medical Centre Hospital, Trollhattan, Sweden

**Keywords:** Head and neck cancer, trismus, preventive exercise, health-related quality of life, radiotherapy

## Abstract

**Objectives:**

The aim of the study was to investigate the effect of preventive exercise therapy on radiation-induced trismus in head and neck cancer.

**Materials and methods:**

Eighty-nine patients (*n* = 89) with newly diagnosed head and neck cancer were included and randomized to either the preventive exercise intervention group (*n* = 45) or the control group. (*n* = 44). The intervention group was instructed to perform jaw opening exercises once daily.

**Results:**

No differences were found in mouth opening capacity between the intervention and the control groups in the first year after oncological treatment. At the 6-month follow-up, the prevalence of trismus was 7% and 19% in the intervention and the control groups, respectively, and at the 12-month follow-up, 7% and 3%, respectively; these differences were not statistically significant.

**Conclusion:**

Preventive exercise was not effective in improving trismus and mouth opening-related outcomes in this randomized study. A surprisingly low prevalence of trismus was seen in both groups in the first year after radiotherapy.

**Clinical relevance:**

There is reason to believe that newer radiotherapy regimens have reduced radiation trismus in HNC. Although prophylactic intervention does not appear to influence mouth opening capacity, it is important to carefully monitor mouth opening in patients undergoing radiotherapy for HNC. Early detection of a reduction in MIO can permit early initiation of exercise interventions for trismus.

## Background

Patients with head and neck cancer (HNC) suffer from various treatment- and tumor-related symptoms during and after oncological treatment. Among these, radiation-induced trismus is particularly burdensome and can lead to difficulties in eating and chewing and is also linked to pain and jaw-related problems. Trismus and dysphagia are symptoms that often persist and can lead to malnutrition and negatively influence the health-related quality of life (HRQoL) of HNC patients (1, 2). The occurrence of trismus is closely related to the radiation dose delivered to the muscles of mastication and adjacent structures. Specifically, the masseter and pterygoid muscles have been identified as crucial risk structures for the development of radiation-induced trismus (3–7).

Exercise interventions for the treatment of radiation-induced trismus have been suggested to be effective in improving mouth opening outcomes in some studies (8, 9). However, few randomized trials have been carried out, and the results from these have not been able to provide any solid evidence to support the beneficial effects of exercise interventions (10). Prehabilitative measures for dysphagia related to HNC have shown promising results in terms of maintaining swallowing function throughout radiotherapy, but few studies have focused on trismus and the results are conflicting (11–14).

The aim of the study was to investigate the effect of preventive exercise therapy on radiation-induced trismus in HNC patients up to one year after treatment. More specifically, the study compared the prevalence of trismus, defined as a maximal interincisal opening (MIO) of ≤ 35 mm, and patient-reported mouth opening problems according to the validated and symptom-specific Gothenburg Trismus Questionnaire in participants randomized to preventive exercise compared to a control group.

## Materials and methods

The present study is a part of a randomized clinical trial investigating preventive interventions for swallowing and jaw related problems in head and neck cancer patients. All study participants were enrolled at Sahlgrenska university Hospital, Sweden during 2019–2022.The protocol and results from the first follow-up at approximately 1 month after radiotherapy have previously been published by the research group (16).

### Study subjects

Adult patients (> 18 years of age) with newly diagnosed HNC scheduled for radiotherapy with curative intent for oropharyngeal, hypopharyngeal, or laryngeal tumors were asked to participate in the study. Exclusion criteria were restricted mouth opening (MIO ≤ 35 mm) or swallowing difficulties before HNC, previous treatment for HNC, inability to perform exercise or to independently fill out questionnaires, and neurological disease with expected swallowing difficulties. Data regarding smoking, alcohol use, marital status, comorbidities, BMI, education, and employment status were collected.

The study participants were randomized to either a preventive exercise intervention group or a control group. Participants in both groups were in contact with a speech-language pathologist on a weekly basis throughout the study period. All participants received information regarding the possible side effects of oncological treatment, including jaw opening problems and swallowing problems. All participants were encouraged to continue to eat and drink for as long as possible during oncological treatment to maintain swallowing function. Data were collected at baseline i.e. before start of oncological treatment, and follow-up visits at 1–2 months after completion of radiotherapy and at 6 and 12 months.

### Oncological treatment

External beam radiation therapy (EBRT) was delivered as volumetric-modulated arc therapy (VMAT). The schedule was moderately accelerated fractionation administered once or twice daily with 2 Gy fractions to a total of 68 Gy with six treatments per week. Most participants also received irradiation to the cervical lymph nodes to a total of 52.7 Gy, given in 1.55 Gy fractions. Most of the participants in the study also received concomitant chemotherapy.

### Randomization procedure

The randomization procedure was performed according to optimal allocation with the Pocock randomization sequence considering sex, age, tumor location, tumor size, jaw opening, and swallowing before the start of oncological treatment. Swallowing difficulties before oncological treatment were determined to be present if the patient responded yes to three or more of the following questions: “Do you have difficulty eating, drinking, swallowing, or cough during meals?”(15).

### Jaw exercise intervention

The preventive exercise group was instructed to perform jaw opening exercises using the Jawtrainer© and swallowing exercises. The jaw opening exercise consisted of:

- Passive jaw stretch using the Jawtrainer© for jaw opening for 30 s, repeated three times.
- Active jaw stretch using the Jawtrainer© for mouth opening against resistance, repeated five times.

Besides these jaw opening exercises, the patients were instructed to perform swallowing exercises according to the Masako maneuver as a part of a larger study on swallowing. The exercise program was to be performed once daily. All participants in the preventive exercise group were instructed to fill out an exercise diary including weekly measurements of mouth opening ability using a mouth opening measurement ruler. The exercise diary assessed the compliance to exercise.

### Endpoints

The primary endpoint of the study was mouth opening measured in millimetres were trismus was defined as MIO ≤ 35 mm (17),. The secondary endpoints were patient-reported mouth opening symptoms according to the Gothenburg Trismus Questionnaire (GTQ2) and the HNC-specific module of the European Organization for Research and Treatment of Cancer Quality of Life Questionnaire (EORTC HN35).

### Patient-reported outcomes

To assess jaw related problems and restricted mouth opening, the revised version of the GTQ2 was used (18, 19). This instrument describes symptoms of jaw-related problems in relation to HRQL. GTQ2 consists of 29 items of which 20 items are divided into four domains; Jaw related problems (8 items), Eating limitations (4 items), Muscular tension (3 items) and Facial pain (5 items). It also includes 3 single items about mouth opening and 6 questions related to any kind of training to improve mouth opening. The GTQ 2 also includes a picture of a face giving the patient the possibility to locate the pain. The domains are scored by calculating the mean of each domain and transforming it to a scale of range 0 to 100, where a maximum score indicates greater symptom burden due to trismus.

HRQoL and patient-reported symptoms were assessed using the European Organization for Research and Treatment of Cancer Quality of Life Questionnaire Core 30 (EORTC C30) (5). All scale scores range from 0 to 100 points. High scores for a functional scale and the global quality of life scale represent a high level of functioning or a high level of global quality of life. Conversely, high scores for a single item represent a high level of symptoms. To further assess HNC-specific symptoms, the EORTC HN35 was used (20).

## Statistical methods

Data are reported as mean and standard deviation, median (range), the number of observations, and percentages. To test differences between the two groups, the following statistical methods were used: Fisher’s exact test for dichotomous variables, the Mantel-Haenszel chi-squared trend test for ordered categorical variables, the chi-squared test for non-ordered categorical variables, the Mann-Whitney U-test for continuous variables that were not normally distributed, and the two-sample t-test for normally distributed continuous variables. Power calculations determined a sample size of 80 patients in two equally sized groups was needed to provide 80% power to detect a clinically relevant difference of 5 mm in jaw opening (SD 5.8). All tests were two-tailed. A p-value of 0.05 was applied. All analyses were performed by using SAS software version 9.4 (SAS Institute Inc., Cary, NC, USA).

## Ethics

The study was approved by the regional ethical review board in Gothenburg, Sweden (number 1151-18/2019 − 00752) and performed in accordance with the Declaration of Helsinki. All participants gave their written informed consent to participate in the study.

## Results

### Study group characteristics

**Fig. 1:**
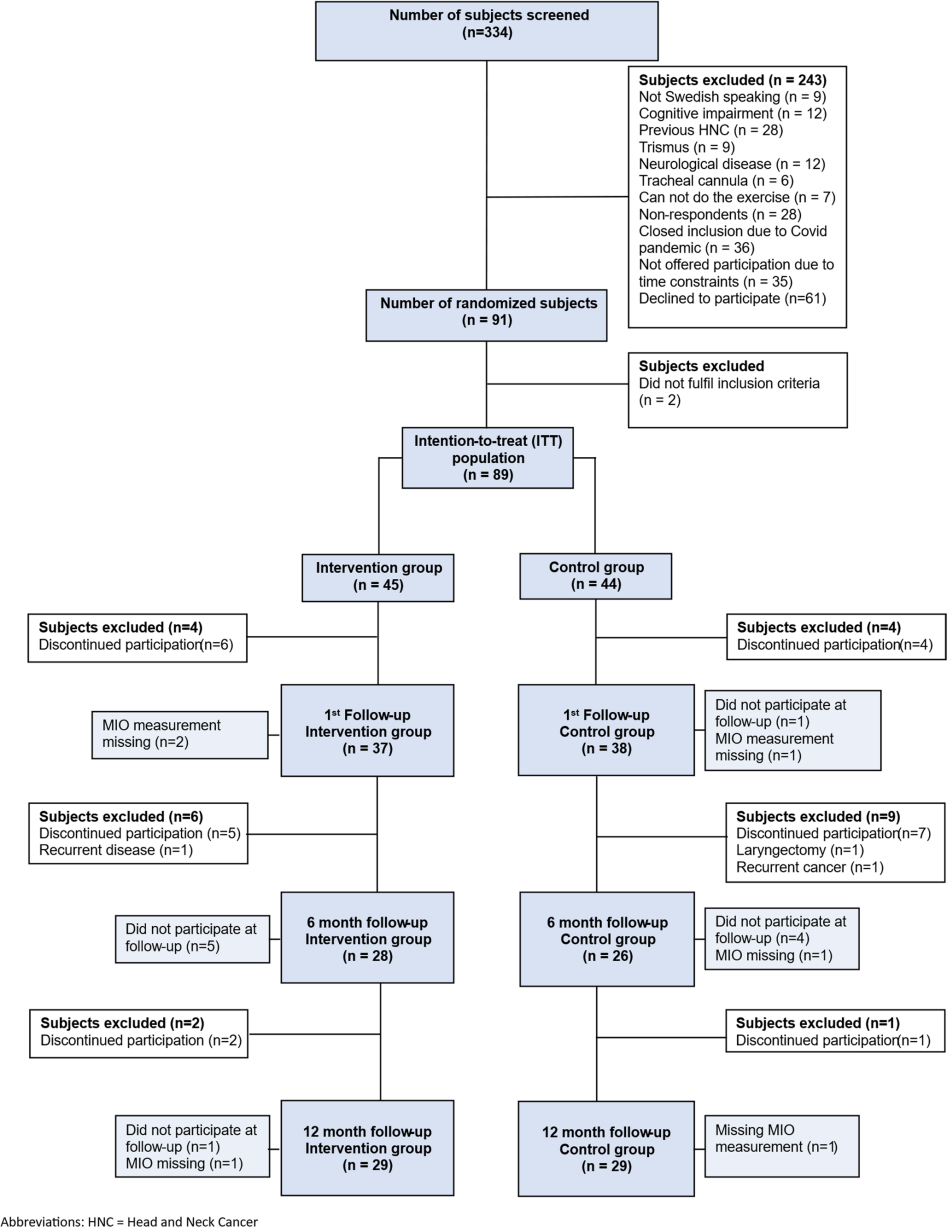
Study flow chart

Eighty-nine patients (*n* = 89) with newly diagnosed HNC were included and randomized to either the preventive exercise intervention group (*n* = 45) or the control group (*n* = 44). At the 12 month follow up 29 patients remained in each group, Fig. 1. The mean age was 64 and 65 years in the control and intervention groups, respectively. The male-to-female ratio was 3:1 in both groups. The most common tumor location was tonsil cancer and base of tongue tumors in both groups. Tonsil cancer constituted more than half of the tumors in this study population. The overall rate of HPV-positive tumors was 88% and 87% in the control and intervention groups, respectively. There were no statistically significant differences in tumor size (T-stage) or stage (TNM stage) or any other baseline variables between the groups, see Table 1. All participants were dentate, with only a few participants using prostheses in the upper or lower jaw.

**Table 1:** Patient characteristics at baseline

	Control *N* = 44	Intervention *N* = 45	*p*-value
Age (years) mean (SD) median (min-max)	63.5 ± 9.2 65 (42–83) *n* = 44	64.5 ± 9.7 64 (43–81) *n* = 45	0.68
Sex			0.81
Male	33 (75.0%)	35 (77.8%)	
Female	11 (25.0%)	10 (22.2%)	
**Occupation**			0.63
Working	15 (34.1%)	16 (35.6%)	
Sick leave	9 (20.5%)	6 (13.3%)	
Retired	20 (45.5%)	22 (48.9%)	
Unemployed	0 (0.0%)	1 (2.2%)	
**Smoking**			0.47
Never smoked	16 (38.1%)	17 (45.9%)	
Stopped smoking > 12 months	17 (40.5%)	11 (29.7%)	
Stopped smoking < 12 months	5 (11.9%)	9 (24.3%)	
Smoking	4 (9.5%)	0 (0.0%)	
Missing	2	8	
**Tumor location**			0.88
Tonsil	25 (56.8%)	25 (55.6%)	
Base of tongue	13 (29.5%)	13 (28.9%)	
Hypopharynx	3 (6.8%)	2 (4.4%)	
Larynx	3 (6.8%)	5 (11.1%)	
**Tumor stage**			0.51
I	23 (52.3%)	23 (51.1%)	
II	2 (4.5%)	5 (11.1%)	
III	12 (27.3%)	15 (33.3%)	
IV	7 (15.9%)	2 (4.4%)	
**T-stage**			0.53
1	6 (13.6%)	10 (22.2%)	
2	23 (52.3%)	20 (44.4%)	
3	2 (4.5%)	4 (8.9%)	
4	13 (29.5%)	11 (24.4%)	
**Oncological treatment**			0.32
Radiotherapy	7 (15.9%)	11 (24.4%)	
Radiochemotherapy	37 (84.1%)	34 (75.6%)	
**Comorbidity ACE-27 score**			0.21
None	20 (45.5%)	28 (62.2%)	
Mild	14 (31.8%)	10 (22.2%)	
Moderate	10 (22.7%)	6 (13.3%)	
Severe	0 (0.0%)	1 (2.2%)	
HPV positive			0.92
No	2 (4.7%)	3 (6.7%)	
Yes	38 (88.4%)	39 (86.7%)	
Not tested	3 (7.0%)	3 (6.7%)	
Missing	1	0	
**Teeth**			0.38
Own teeth	40 (93.0%)	38 (95.0%)	
Prosthesis lower jaw	3 (7.0%)	1 (2.5%)	
Prosthesis upper jaw	0 (0.0%)	1 (2.5%)	
Missing	1	5	

### Mouth opening ability

There were no differences in mouth opening capacity as measured by the mean MIO between the intervention and control groups at baseline or any follow-up occasion in the first year after oncological treatment. Both groups experienced a decrease in mouth opening capacity from baseline in the first year. At the 6-month follow-up, the prevalence of trismus (MIO ≤ 35 mm) was 7% and 19% in the intervention and control groups, respectively, and at the 12-month follow-up, 7% and 3%, respectively; these differences were not statistically significant, Table 2.

**Table 2:** Mouth opening capacity (maximal interincisal opening) in head and neck cancer patients before and after oncological treatment in the intervention and the control group

	Baseline			1st follow up			6 months			12 months		
	Intervention *n* = 44	Control *n* = 45	*p*-value	Intervention *n* = 37	Control *n* = 38	*p*-value	Intervention *n* = 28	Control *n* = 26	*P*	Intervention *n* = 29	Control *n* = 29	*p*-value
MIO mean SD median (min-max)(mm)	49.7 ± 6.4 50.0 (39.0–67.0	49.7 ± 6.4 49.0 (37.0–64.0)	0.82	45.1 ± 7.3 45.0 (30.0–68.0)	42.1 ± 9.6 41.5 (20.0–61.0)	0.15	44.0 ± 7.2 42.0 (28.0–65.0)	43.8 ± 9.3 43.5 (13.0–55.0)	0.65	46.3 ± 8.0 44.0 (32.0–70.0)	46.1 ± 7.4 45.0 (28.0–60.0)	0.91
Change MIO compared to baseline				-4.2 ± 5.2 -4.0 (-19.0–6.0)	-7.0 ± 8.7 -6.0 (-31.0–11.0)	0.13	-5.4 ± 6.5 -3.0 (-26.0–3.0)	-6.0 ± 8.4 -5.0 (-37.0–5.0)	0.94	-3.8 ± 5.6 -3.0 (-22.0–8.0)	-3.6 ± 5.8 -2.0 (-22.0–8.0)	0.76
MIO ≤ 35 mm n(%)	0	0	-	2 (5.6%)	7 (18.4%)	0.15	2 (7.1%)	5 (19.2%)	0.24	2 (6.9%)	1 (3.4%)	1.00
Overall prevalence n(%)						9 (12.0%)			7 (13.0%)			3 (5.2%)

### Adherence to exercise

**Fig. 2:**
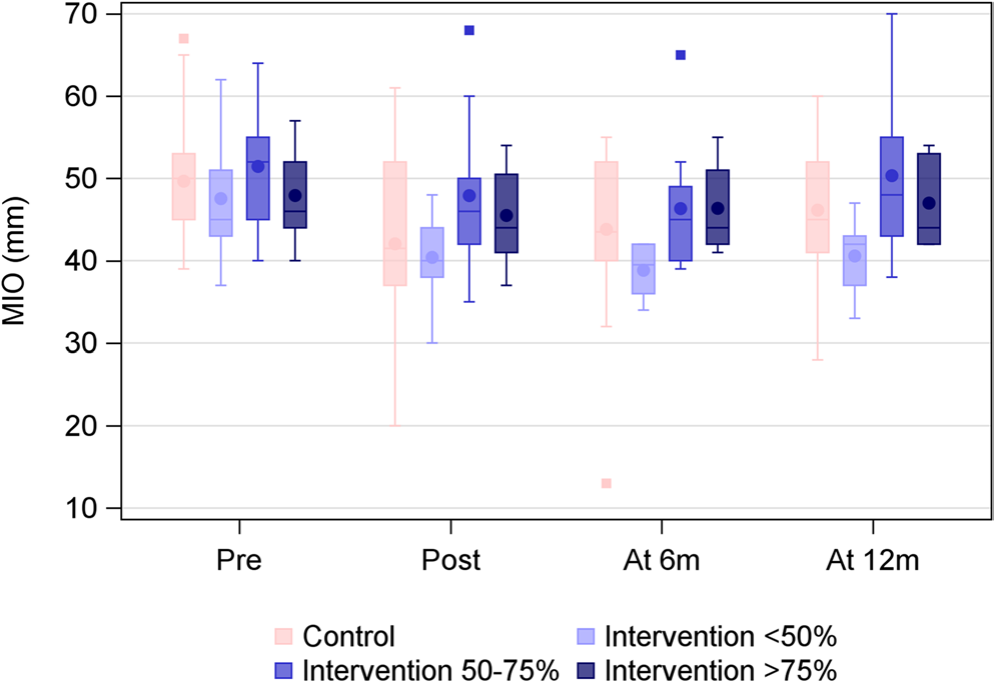
MIO (mm) per treatment group and adherence over time

Overall, 62% of participants in the intervention group complied with the recommended exercise program. Among the participants in this group, there were no pronounced differences in MIO based on the degree of adherence to the exercise intervention, Table 3. However, a trend toward reduced mouth opening capacity was noted in the group with the lowest degree of adherence, Fig. 2.

**Table 3:** Adherence to exercise in the intervention group and the relation to maximal interincisal opening during the first year of follow-up. MIO presented with mean SD median (min-max)(mm)

Exercise adherence *n* (%)	MIO (mm) Baseline	MIO (mm) 1st follow up	MIO (mm) 6 months	MIO (mm) 12 months
< 50%	47.5 ± 6.7 45.0 (37.0–62.0) *n* = 11	40.4 ± 5.2 40.0 (30.0–48.0) *n* = 10	38.8 ± 3.3 39.5 (34.0–42.0) *n* = 6	40.6 ± 4.6 42.0 (33.0–47.0) *n* = 7
50–75%	51.5 ± 7.4 52.0 (40.0–64.0) *n* = 11	47.9 ± 9.2 46.0 (35.0–68.0) *n* = 11	46.3 ± 8.3 45.0 (39.0–65.0) *n* = 9	50.3 ± 11.2 48.0 (38.0–70.0) *n* = 6
> 75%	47.9 ± 5.2 46.0 (40.0–57.0) *n* = 13	45.5 ± 5.9 44.0 (37.0–54.0) *n* = 12	46.4 ± 5.1 44.0 (41.0–55.0) *n* = 11	47.0 ± 5.3 44.0 (42.0–54.0) *n* = 11
p-value	0.30	0.06	0.050	0.054
Mean adherence				
Passive jaw exercise proportion of recommended dose	0.62 ± 0.31 0.64 (0.00–1.00) *n* = 35			
Active jaw exercise proportion of recommended dose	0.62 ± 0.31 0.64 (0.00–1.00) *n* = 35			

## Patient-reported outcomes

### Gothenburg trismus questionnaire (GTQ2)

Regarding jaw-related problems, eating limitations, muscular tension, and facial pain, we found no differences between the intervention and control groups at baseline or any of the follow-up occasions. There was a tendency toward a higher prevalence of facial pain at baseline in the intervention group, which was not statistically significant, Table 4. Differences in the GTQ score between the two groups were not significant at any of the follow-up occasions. Changes in the GTQ score over time within each group were also not statistically significant.

**Table 4:** Gothenburg Trismus Questionnaire score in the intervention group (I) and the control group (C)

GTQ Mean (CI)	Baseline			1st follow up			6 months			12 months		
	I, *n* = 39	C, *n* = 43	*p*-value	I, *n* = 37	C, *n* = 37	*p*-value	I, *n* = 35	C, *n* = 32	*p*-value	I, *n* = 32	C, *n* = 30	*p*-value
Jaw-related problems	10.6 ± 17.0 0.0 (0.0–65.6)	13.0 ± 33.4 0.0 (0.0–203.1)	0.56	24.6 ± 20.0 21.9 (0.0–71.9)	17.1 ± 18.0 12.5 (0.0–62.5)	0.08	18.3 ± 18.9 15.6 (0.0–78.1)	14.0 ± 21.0 4.7 (0.0–84.4)	0.07	13.6 ± 15.6 7.8 (0.0–53.1)	10.5 ± 16.3 3.1 (0.0–81.3)	0.33
Eating limitations	9.5 ± 20.2 0.0 (0.0–81.3)	3.6 ± 9.3 0.0 (0.0–43.8)	0.37	22.3 ± 26.5 12.5 (0.0–93.8)	18.8 ± 27.3 6.3 (0.0–100.0)	0.39	10.0 ± 14.8 6.3 (0.0–50.0)	15.4 ± 26.8 6.3 (0.0–100.0)	0.75	10.4 ± 13.3 3.1 (0.0–43.8)	11.0 ± 19.7 0.0 (0.0–75.0)	0.61
Muscular tension	11.5 ± 14.9 8.3 (0.0–50.0)	11.2 ± 17.0 0.0 (0.0–83.3)	0.71	16.7 ± 16.1 16.7 (0.0–50.0)	15.7 ± 18.7 8.3 (0.0–75.0)	0.58	19.3 ± 21.0 16.7 (0.0–66.7)	12.4 ± 15.6 8.3 (0.0–50.0)	0.23	17.4 ± 20.5 8.3 (0.0–75.0)	12.8 ± 18.9 0.0 (0.0–66.7)	0.33
GTQ facial pain	10.3 ± 20.0 0.0 (0.0–80.0)	4.2 ± 12.7 0.0 (0.0–65.0)	0.07	9.0 ± 15.7 0.0 (0.0–50.0)	7.8 ± 13.8 0.0 (0.0–50.0)	0.88	7.3 ± 13.1 0.0 (0.0–50.0)	6.2 ± 13.7 0.0 (0.0–62.5)	0.48	3.9 ± 8.8 0.0 (0.0–30.0)	3.2 ± 11.4 0.0 (0.0–60.0)	0.53
GTQ How limited are you in your ability to open mouth	7.9 ± 17.5 0.0 (0.0–75.0)	2.3 ± 9.2 0.0 (0.0–50.0)	0.07	19.6 ± 22.2 25.0 (0.0–75.0)	15.3 ± 21.8 0.0 (0.0–75.0)	0.32	12.1 ± 19.5 0.0 (0.0–75.0)	15.6 ± 24.4 0.0 (0.0–100.0)	0.61	7.0 ± 13.1 0.0 (0.0–50.0)	9.2 ± 21.3 0.0 (0.0–100.0)	0.96
GTQ Limitation interfering with social	5.3 ± 17.6 0.0 (0.0–100.0)	2.3 ± 9.2 0.0 (0.0–50.0)	0.37	11.5 ± 17.3 0.0 (0.0–50.0)	10.4 ± 18.3 0.0 (0.0–50.0)	0.63	5.9 ± 13.8 0.0 (0.0–50.0)	5.5 ± 15.2 0.0 (0.0–75.0)	0.82	3.9 ± 9.2 0.0 (0.0–25.0)	1.7 ± 9.3 0.0 (0.0–50.0)	0.14
GTQ Limitation ability to work	6.1 ± 19.0 0.0 (0.0–100.0)	1.2 ± 7.6 0.0 (0.0–50.0)	0.07	12.5 ± 20.3 0.0 (0.0–75.0)	9.7 ± 18.2 0.0 (0.0–50.0)	0.43	5.9 ± 13.8 0.0 (0.0–50.0)	2.3 ± 7.4 0.0 (0.0–25.0)	0.31	2.3 ± 7.4 0.0 (0.0–25.0)	0.0 ± 0.0 0.0 (0.0–0.0)	0.11

* Domains and single item scores range from 0 to 100, where 100 indicates the maximum amount of symptoms and 0 is equal to no symptoms. GTQ, Gothenburg Trismus Questionnaire. CI, 95% confidence interval.

### EORTC QLQ HN35 and C30

No statistically significant differences were found between the two groups on any item of the EORTC HN35 questionnaire except for “Felt ill” at the first follow-up. The most pronounced increases in the prevalence of symptoms were seen in both groups for “Dry mouth” and “Sticky saliva” at the 6- and 12-month follow-ups, Table 5. No differences were found between the groups regarding the EORTC QLQ C30 questionnaire except for “Insomnia” at the 12-month follow-up and “Appetite loss” at the first follow-up, Table 6.

**Table 5:** EORTC QLQ HN35-score for HNC patients in the intervention group (I) and the control group (C)

	Baseline			1st follow up			6 months			12 months		
	I, *n* = 40 Mean (CI)	C, *n* = 43 Mean (CI)	*p*-value	I, *n*= Mean (CI)	C, *n*= Mean (CI)	*p*-value	I, *n* = 35 Mean (CI)	C, *n* = 32 Mean (CI)	*p*-value	I, *n* = 32 Mean (CI)	C, *n* = 30 Mean (CI)	*p*-value
Pain	20.8 ± 25.5 12.5 (0.0–100.0) *n* = 40	18.2 ± 22.9 8.3 (0.0–91.7) *n* = 43	0.68	35.4 ± 28.6 33.3 (0.0–100.0) *n* = 33	22.9 ± 18.6 16.7 (0.0–83.3) *n* = 31	0.11	19.5 ± 21.8 8.3 (0.0–66.7) *n* = 35	14.8 ± 21.9 4.2 (0.0–91.7) *n* = 32	0.21	14.8 ± 24.3 0.0 (0.0–75.0) *n* = 32	10.1 ± 17.9 0.0 (0.0–66.7) *n* = 29	0.58
Swallowing	11.9 ± 21.5 0.0 (0.0–83.3) *n* = 40	10.3 ± 18.8 0.0 (0.0–66.7) *n* = 43	0.59	33.8 ± 25.1 33.3 (0.0–91.7) *n* = 33	23.9 ± 25.3 11.1 (0.0–91.7) *n* = 31	0.09	18.8 ± 21.2 16.7 (0.0–83.3) *n* = 35	14.1 ± 18.4 8.3 (0.0–75.0) *n* = 32	0.37	18.2 ± 22.9 12.5 (0.0–100.0) *n* = 32	15.6 ± 20.8 8.3 (0.0–83.3) *n* = 30	0.48
Senses	13.3 ± 22.4 0.0 (0.0–83.3) *n* = 40	10.1 ± 20.6 0.0 (0.0–100.0) *n* = 43	0.53	42.9 ± 30.1 50.0 (0.0–100.0) *n* = 33	36.1 ± 23.9 33.3 (0.0–100.0) *n* = 31	0.26	30.0 ± 24.2 33.3 (0.0–83.3) *n* = 35	28.1 ± 25.2 16.7 (0.0–83.3) *n* = 32	0.72	29.7 ± 28.6 33.3 (0.0–100.0) *n* = 32	24.4 ± 20.4 25.0 (0.0–66.7) *n* = 30	0.67
Speech	10.8 ± 14.9 0.0 (0.0–55.6) *n* = 37	8.5 ± 10.9 5.6 (0.0–44.4) *n* = 42	0.83	20.5 ± 22.4 11.1 (0.0–88.9) *n* = 33	16.5 ± 16.7 11.1 (0.0–66.7) *n* = 31	0.62	17.1 ± 21.1 11.1 (0.0–100.0) *n* = 35	11.8 ± 16.4 11.1 (0.0–66.7) *n* = 32	0.29	13.5 ± 19.7 11.1 (0.0–88.9) *n* = 32	10.0 ± 10.7 11.1 (0.0–33.3) *n* = 30	0.90
Social eating	9.7 ± 17.5 0.0 (0.0–83.3) *n* = 37	9.5 ± 14.2 0.0 (0.0–50.0) *n* = 42	0.84	31.6 ± 24.5 33.3 (0.0–100.0) *n* = 33	24.1 ± 22.0 23.6 (0.0–100.0) *n* = 30	0.16	20.2 ± 20.7 16.7 (0.0–75.0) *n* = 35	17.5 ± 26.0 8.3 (0.0–100.0) *n* = 31	0.35	16.4 ± 22.5 8.3 (0.0–83.3) *n* = 32	16.8 ± 24.3 8.3 (0.0–100.0) *n* = 30	0.83
Social contact	5.3 ± 15.2 0.0 (0.0–80.0) *n* = 37	14.8 ± 61.2 0.0 (-13.3 -66.7) *n* = 42	0.56	12.1 ± 17.4 0.0 (0.0–73.3) *n* = 33	12.0 ± 16.6 6.7 (0.0–53.3) *n* = 31	0.86	8.0 ± 13.5 0.0 (0.0–60.0) *n* = 35	7.7 ± 18.2 0.0 (0.0–80.0) *n* = 32	0.22	9.2 ± 22.6 0.0 (0.0–80.0) *n* = 32	5.6 ± 16.3 0.0 (0.0–60.0) *n* = 30	0.39
Sexuality	34.3 ± 36.5 33.3 (0.0–100.0) *n* = 36	23.4 ± 34.1 0.0 (0.0–100.0) *n* = 42	0.13	51.0 ± 36.6 66.7 (0.0–100.0) *n* = 32	41.4 ± 42.8 33.3 (0.0–100.0) *n* = 31	0.35	31.9 ± 36.5 33.3 (0.0–100.0) *n* = 35	31.8 ± 37.7 16.7 (0.0–100.0) *n* = 32	0.96	31.7 ± 38.3 16.7 (0.0–100.0) *n* = 31	32.2 ± 35.1 16.7 (0.0–100.0) *n* = 29	0.82
Teeth	15.8 ± 29.2 0.0 (0.0–100.0) *n* = 40	11.6 ± 25.1 0.0 (0.0–100.0) *n* = 43	0.40	9.1 ± 22.5 0.0 (0.0–100.0) *n* = 33	11.8 ± 25.2 0.0 (0.0–100.0) *n* = 31	0.65	7.6 ± 21.5 0.0 (0.0–100.0) *n* = 35	9.4 ± 22.8 0.0 (0.0–100.0) *n* = 32	0.65	13.5 ± 29.2 0.0 (0.0–100.0) *n* = 32	7.8 ± 22.6 0.0 (0.0–100.0) *n* = 30	0.38
Opening mouth	10.8 ± 24.3 0.0 (0.0–100.0) *n* = 40	5.4 ± 14.4 0.0 (0.0–66.7) *n* = 43	0.40	17.2 ± 20.6 0.0 (0.0–66.7) *n* = 33	15.1 ± 20.8 0.0 (0.0–66.7) *n* = 31	0.63	14.3 ± 21.8 0.0 (0.0–100.0) *n* = 35	19.8 ± 25.2 0.0 (0.0–100.0) *n* = 32	0.35	11.5 ± 20.1 0.0 (0.0–66.7) *n* = 32	16.7 ± 24.4 0.0 (0.0–100.0) *n* = 30	0.36
Dry mouth	16.7 ± 30.2 0.0 (0.0–100.0) *n* = 40	13.2 ± 26.4 0.0 (0.0–100.0) *n* = 43	0.63	58.6 ± 36.4 66.7 (0.0–100.0) *n* = 33	58.1 ± 31.0 66.7 (0.0–100.0) *n* = 31	0.89	58.1 ± 29.5 66.7 (0.0–100.0) *n* = 35	63.5 ± 25.9 66.7 (0.0–100.0) *n* = 32	0.36	59.4 ± 29.0 66.7 (0.0–100.0) *n* = 32	53.3 ± 34.6 33.3 (0.0–100.0) *n* = 30	0.38
Sticky saliva	17.9 ± 29.5 0.0 (0.0–100.0) *n* = 39	13.2 ± 24.3 0.0 (0.0–100.0) *n* = 43	0.52	59.6 ± 36.1 66.7 (0.0–100.0) *n* = 33	60.2 ± 34.9 66.7 (0.0–100.0) *n* = 31	0.96	46.7 ± 34.5 33.3 (0.0–100.0) *n* = 35	39.6 ± 31.0 33.3 (0.0–100.0) *n* = 32	0.41	43.7 ± 38.3 33.3 (0.0–100.0) *n* = 32	46.7 ± 35.7 33.3 (0.0–100.0) *n* = 30	0.70
Coughed	18.3 ± 25.0 0.0 (0.0–100.0) *n* = 40	17.8 ± 22.2 0.0 (0.0–100.0) *n* = 43	0.90	24.2 ± 25.4 33.3 (0.0–66.7) *n* = 33	33.3 ± 27.2 33.3 (0.0–100.0) *n* = 31	0.19	32.4 ± 34.8 33.3 (0.0–100.0) *n* = 35	19.8 ± 18.7 33.3 (0.0–66.7) *n* = 32	0.24	33.3 ± 30.5 33.3 (0.0–100.0) *n* = 32	24.4 ± 27.6 33.3 (0.0–100.0) *n* = 30	0.24
Felt ill	19.3 ± 29.0 0.0 (0.0–100.0) *n* = 40	18.6 ± 26.5 0.0 (0.0–100.0) *n* = 43	0.97	35.4 ± 31.1 33.3 (0.0–100.0) *n* = 33	19.4 ± 24.0 0.0 (0.0–100.0) *n* = 31	**0.032**	13.3 ± 18.4 0.0 (0.0–66.7) *n* = 35	10.4 ± 23.1 0.0 (0.0–100.0) *n* = 32	0.24	10.4 ± 23.1 0.0 (0.0–100.0) *n* = 32	8.9 ± 26.2 0.0 (0.0–100.0) *n* = 30	0.44
Pain killers	43.2 ± 50.2 0.0 (0.0–100.0) *n* = 37	40.5 ± 49.7 0.0 (0.0–100.0) *n* = 42	0.81	69.7 ± 46.7 100.0 (0.0–100.0) *n* = 33	61.3 ± 49.5 100.0 (0.0–100.0) *n* = 31	0.49	22.9 ± 42.6 0.0 (0.0–100.0) *n* = 35	18.8 ± 39.7 0.0 (0.0–100.0) *n* = 32	0.69	15.6 ± 36.9 0.0 (0.0–100.0) *n* = 32	20.0 ± 40.7 0.0 (0.0–100.0) *n* = 30	0.66

* Score range 0-100 points. High scores for a single item represent a high level of symptoms. CI, 95% confidence interval.

**Table 6:** EORTC QLQ C30 score for HNC patients in the intervention group (I) and the control group (C)

Functional scales	Baseline			1st follow up			6 months			12 months		
	I, *n* = 40 Mean (CI)	C, *n* = 43 Mean (CI)	*p*-value	I, *n* = 33 Mean (CI)	C, *n* = 31 Mean (CI)	*p*-value	I, *n* = 35 Mean (CI)	C, *n* = 32 Mean (CI)	*p*-value	I, *n* = 32 Mean (CI)	C, *n* = 30 Mean (CI)	*p*-value
Physical Functioning	89.2 ± 19.4 100.0 (33.3–100.0) *n* = 40	91.8 ± 12.6 100.0 (46.7–100.0) *n* = 43	0.69	78.8 ± 19.7 86.7 (26.7–100.0) *n* = 33	77.8 ± 17.6 80.0 (26.7–100.0) *n* = 31	0.67	85.3 ± 17.1 86.7 (33.3–100.0) *n* = 35	89.6 ± 13.3 93.3 (53.3–100.0) *n* = 32	0.33	89.2 ± 19.1 100.0 (40.0–100.0) *n* = 32	89.3 ± 12.3 93.3 (60.0–100.0) *n* = 30	0.32
Role Functioning	74.2 ± 36.2 100.0 (0.0–100.0) *n* = 40	82.2 ± 26.1 100.0 (33.3–100.0) *n* = 43	0.37	49.5 ± 36.0 50.0 (0.0–100.0) *n* = 33	55.4 ± 31.1 50.0 (0.0–100.0) *n* = 31	0.58	76.2 ± 31.4 100.0 (0.0–100.0) *n* = 35	84.4 ± 23.9 100.0 (33.3–100.0) *n* = 32	0.30	85.0 ± 24.1 100.76.2 ± 31.4 100.0 (0.0–100.0) *n* = 35(0.0–100.0) *n* = 30	78.1 ± 30.1 100.0 (0.0–100.0) *n* = 32	0.45
Emotional Functioning	73.5 ± 24.8 83.3 (16.7–100.0) *n* = 40	71.9 ± 24.2 75.0 (0.0–100.0) *n* = 43	0.70	70.7 ± 26.1 75.0 (0.0–100.0) *n* = 33	75.8 ± 24.8 83.3 (16.7–100.0) *n* = 31	0.48	82.4 ± 25.5 91.7 (0.0–100.0) *n* = 35	81.5 ± 19.7 91.7 (33.3–100.0) *n* = 32	0.50	84.4 ± 25.5 100.0 (0.0–100.0) *n* = 32	79.7 ± 26.0 91.7 (16.7–100.0) *n* = 30	0.46
Cognitive Functioning	80.4 ± 26.7 100.0 (16.7–100.0) *n* = 40	82.9 ± 25.6 83.3 (0.0–100.0) *n* = 43	0.89	72.7 ± 29.1 83.3 (0.0–100.0) *n* = 33	82.3 ± 23.1 83.3 (0.0–100.0) *n* = 31	0.23	82.4 ± 23.9 83.3 (16.7–100.0) *n* = 35	90.1 ± 15.2 100.0 (33.3–100.0) *n* = 32	0.23	81.3 ± 28.3 100.0 (0.0–100.0) *n* = 32	82.8 ± 24.9 91.7 (16.7–100.0) *n* = 30	0.95
Social Functioning	77.9 ± 27.6 83.3 (0.0–100.0) *n* = 40	84.1 ± 21.2 100.0 (33.3–100.0) *n* = 43	0.37	64.1 ± 28.3 66.7 (0.0–100.0) *n* = 33	62.9 ± 33.8 66.7 (0.0–100.0) *n* = 31	0.99	76.7 ± 32.6 100.0 (0.0–100.0) *n* = 35	81.3 ± 29.6 100.0 (0.0–100.0) *n* = 32	0.48	80.7 ± 30.3 100.0 (0.0–100.0) *n* = 32	90.0 ± 16.7 100.0 (33.3–100.0) *n* = 30	0.38
Global QL	60.6 ± 24.1 62.5 (16.7–100.0) *n* = 40	63.6 ± 26.0 66.7 (8.3–100.0) *n* = 43	0.49	55.1 ± 20.7 50.0 (16.7–100.0) *n* = 33	55.9 ± 22.6 58.3 (0.0–91.7) *n* = 31	0.70	67.4 ± 24.1 75.0 (16.7–100.0) *n* = 35	70.6 ± 22.0 70.8 (16.7–100.0) *n* = 32	0.67	77.9 ± 19.0 83.3 (25.0–100.0) *n* = 32	67.2 ± 26.7 79.2 (0.0–100.0) *n* = 30	0.13
**Symptoms**												
Fatigue	32.5 ± 30.2 33.3 (0.0–100.0) *n* = 40	30.5 ± 28.2 22.2 (0.0–88.9) *n* = 43	0.82	49.5 ± 28.3 55.6 (0.0–100.0) *n* = 33	45.9 ± 23.6 44.4 (11.1–100.0) *n* = 31	0.51	31.4 ± 29.0 33.3 (0.0–100.0) *n* = 35	27.6 ± 26.8 22.2 (0.0–88.9) *n* = 32	0.55	27.4 ± 31.7 22.2 (0.0–100.0) *n* = 32	24.1 ± 25.1 11.1 (0.0–88.9) *n* = 30	0.94
Nausea	6.2 ± 18.0 0.0 (0.0–100.0) *n* = 40	7.0 ± 16.8 0.0 (0.0–66.7) *n* = 43	1.00	10.1 ± 16.1 0.0 (0.0–66.7) *n* = 33	9.4 ± 21.3 0.0 (0.0–100.0) *n* = 30	0.34	2.4 ± 7.2 0.0 (0.0–33.3) *n* = 35	4.2 ± 13.4 0.0 (0.0–50.0) *n* = 32	0.90	6.8 ± 21.5 0.0 (0.0–100.0) *n* = 32	2.8 ± 9.9 0.0 (0.0–50.0) *n* = 30	0.71
Pain	23.8 ± 31.3 8.3 (0.0–100.0) *n* = 40	18.2 ± 25.4 0.0 (0.0–83.3) *n* = 43	0.60	34.3 ± 32.0 33.3 (0.0–100.0) *n* = 33	29.6 ± 29.7 33.3 (0.0–100.0) *n* = 31	0.56	20.5 ± 28.3 16.7 (0.0–100.0) *n* = 35	14.1 ± 22.0 0.0 (0.0–100.0) *n* = 32	0.37	16.1 ± 28.2 0.0 (0.0–100.0) *n* = 32	10.0 ± 20.3 0.0 (0.0–83.3) *n* = 30	0.57
Dyspnea	21.7 ± 29.8 0.0 (0.0–100.0) *n* = 40	19.4 ± 24.4 0.0 (0.0–100.0) *n* = 43	0.99	39.4 ± 33.8 33.3 (0.0–100.0) *n* = 33	31.1 ± 27.6 33.3 (0.0–100.0) *n* = 30	0.37	20.0 ± 23.2 33.3 (0.0–100.0) *n* = 35	16.7 ± 23.9 0.0 (0.0–66.7) *n* = 32	0.43	19.8 ± 27.9 0.0 (0.0–100.0) *n* = 32	21.1 ± 25.5 0.0 (0.0–66.7) *n* = 30	0.71
Insomnia	32.5 ± 33.3 33.3 (0.0–100.0) *n* = 40	24.0 ± 31.1 0.0 (0.0–100.0) *n* = 43	0.19	29.2 ± 33.6 33.3 (0.0–100.0) *n* = 32	34.4 ± 30.4 33.3 (0.0–100.0) *n* = 31	0.39	29.5 ± 34.1 33.3 (0.0–100.0) *n* = 35	19.8 ± 25.2 0.0 (0.0–66.7) *n* = 32	0.30	31.3 ± 33.8 33.3 (0.0–100.0) *n* = 32	14.4 ± 24.3 0.0 (0.0–100.0) *n* = 30	**0.032**
Appetite loss	15.8 ± 32.9 0.0 (0.0–100.0) *n* = 40	17.8 ± 30.3 0.0 (0.0–100.0) *n* = 43	0.53	43.4 ± 35.8 33.3 (0.0–100.0) *n* = 33	24.4 ± 28.9 16.7 (0.0–100.0) *n* = 30	**0.035**	24.8 ± 33.7 0.0 (0.0–100.0) *n* = 35	21.9 ± 30.1 0.0 (0.0–100.0) *n* = 32	0.82	22.9 ± 33.3 0.0 (0.0–100.0) *n* = 32	12.6 ± 25.8 0.0 (0.0–100.0) *n* = 29	0.17
Constipation	16.7 ± 26.1 0.0 (0.0–100.0) *n* = 40	12.4 ± 21.8 0.0 (0.0–66.7) *n* = 43	0.42	28.3 ± 29.0 33.3 (0.0–100.0) *n* = 33	18.3 ± 25.6 0.0 (0.0–100.0) *n* = 31	0.13	14.3 ± 25.9 0.0 (0.0–100.0) *n* = 35	15.1 ± 27.0 0.0 (0.0–100.0) *n* = 31	0.84	15.6 ± 31.7 0.0 (0.0–100.0) *n* = 32	7.8 ± 16.8 0.0 (0.0–66.7) *n* = 30	0.53
Diarrhea	2.5 ± 8.9 0.0 (0.0–33.3) *n* = 40	8.5 ± 16.4 0.0 (0.0–66.7) *n* = 43	0.052	15.2 ± 26.5 0.0 (0.0–100.0) *n* = 33	7.5 ± 14.2 0.0 (0.0–33.3) *n* = 31	0.36	10.5 ± 23.9 0.0 (0.0–100.0) *n* = 35	5.4 ± 19.4 0.0 (0.0–100.0) *n* = 31	0.26	4.2 ± 11.2 0.0 (0.0–33.3) *n* = 32	12.2 ± 23.9 0.0 (0.0–100.0) *n* = 30	0.15
Financial Problems	8.5 ± 18.3 0.0 (0.0–66.7) *n* = 39	15.5 ± 27.6 0.0 (0.0–100.0) *n* = 43	0.28	13.1 ± 28.8 0.0 (0.0–100.0) *n* = 33	14.0 ± 28.3 0.0 (0.0–100.0) *n* = 31	0.74	5.7 ± 12.7 0.0 (0.0–33.3) *n* = 35	9.4 ± 21.1 0.0 (0.0–66.7) *n* = 32	0.74	8.3 ± 20.7 0.0 (0.0–100.0) *n* = 32	10.0 ± 19.9 0.0 (0.0–66.7) *n* = 30	0.65

## Discussion

In this randomized study, a preventive exercise intervention program to avoid restricted mouth opening after radiotherapy for HNC was not proven to be effective and did not reduce the incidence of trismus or alter mouth opening capacity as measured by the MIO relative to a control group that received standard of care… The prevalence of trismus was surprisingly low in both groups in the first year after radiotherapy. We could not see any corresponding reduction in symptom burden for other common radiation-related symptoms, such as dry mouth and sticky saliva.

As expected, mouth opening capacity decreased after radiotherapy in both groups. The prevalence of trismus was higher in the control group both at the first follow-up and the 6-month follow-up. Moreover, over time, there was a tendency toward better mouth opening capacity in participants with a high degree of adherence to the exercise program at the start of the study. While this could indicate a positive effect of the preventive exercise intervention, these findings were not statistically robust. Other studies on prophylactic intervention for trismus have, likewise, not been able to show any beneficial effects of exercise intervention in HNC (12–14).

Interestingly, the prevalence of trismus was low in both groups in this study. A meta-analysis performed in 2019, including 2786 HNC patients from 15 studies, estimated an average trismus prevalence of 44% at 6 months and 32% at 12 months post-radiotherapy (21). In contrast, the corresponding figures in the current study were 13% and 5.2% at 6- and 12-months, respectively. It is well known that the prevalence of trismus is related to off-target irradiation of the muscles of mastication and adjacent structures; specifically, the pterygoids and the masseter muscles have been pointed out as risk structures (4, 6, 22–25). There is reason to believe that current radiotherapy regimens, particularly the introduction of VMAT, have reduced radiation doses delivered to the structures that are critical for the development of trismus post-radiotherapy (26). Compared with three-dimensional conformal radiation therapy (3DCRT), intensity-modulated radiation therapy (IMRT) can decrease the occurrence and severity of trismus (27, 28). In a review article, Bensadoun et al. calculated a weighted prevalence of trismus of 25% in HNC populations treated with 3DCRT and 5% with IMRT (27). In the present study, all HNC patients were treated with VMAT reflecting the current standards of treatment at the study site. Another Study have indicated that VMAT is superior to IMRT in terms of delivering lower irradiation doses to established risk organs in HNC (29). VMAT may lower the prevalence of trismus even further in the future.

Although prophylactic intervention does not appear to influence mouth opening capacity, it is important to carefully monitor this function in patients undergoing radiotherapy for HNC. Early detection of a reduction in MIO can permit early initiation of exercise interventions for trismus. Furthermore, individually tailored intervention programs could be created for HNC patients to maintain and protect mouth opening and swallowing functions during and after oncological treatment. Such interventions could take into account knowledge of individual risk factors for trismus, such as tumor localization (7, 30), comorbidities (23), and radiotherapy dose to specific anatomical risk structures (4, 6, 23, 24).

The scientific evidence to support prehabilitation measures for trismus in HNC is still lacking, and there are no solid data to affirm its effectiveness. Nevertheless, some studies indicate that trismus interventions can provide significant benefits when initiated early after the onset of symptoms (8). Hence, it is important to monitor mouth opening capacity on a regular basis to detect trismus in patients undergoing radiotherapy. Altogether, the results highlight the need to carefully navigate between different add-on interventions to manage symptoms for HNC patients who face a significant symptom burden related to oncological treatment.

### Strengths and limitations

The strengths of the study are the prospective randomized design with the use of both objective and subjective patient-reported outcomes as endpoints as well as the high degree of adherence to the exercise intervention. In addition, the intervention group did not differ from the control group in terms of disease burden, comorbidity, or treatment regimen. The follow-up period of 12 months is relevant to the development of radiation-induced trismus as this complication typically develops during the first year post-radiotherapy. A study limitation is that all patients were treated at one HNC center following current oncological treatment guidelines, which could make results difficult to generalize to other settings and populations.

## Conclusion

Preventive exercise intervention to avoid restricted mouth opening after radiotherapy for HNC was not proven to be effective in this randomized study. It is important to monitor the mouth opening capacity in patients undergoing radiotherapy for HNC to detect a reduction in MIO and to initiate early exercise intervention in the case of detected trismus. Exercise interventions should ideally be tailored in the future for each HNC patient depending on individual risk factors.

## Data Availability

The data that support the findings of this study are available from the corresponding author upon reasonable request.

## Declarations

### Clinical trial registration

Registered at https://clinicaltrials.gov/

### Registration number

NCT04005521

### Date of registration

2019-07-02
